# Dimorphic cells: a common feature throughout the low nuclear grade breast neoplasia spectrum

**DOI:** 10.1007/s00428-022-03438-w

**Published:** 2022-11-15

**Authors:** Mirthe de Boer, Paul J. van Diest

**Affiliations:** Department of Pathology, University Medical Center Utrecht, Heidelberglaan 100, 3584 CX Utrecht, Netherlands

**Keywords:** Breast cancer, Precursor lesions, Atypia, Columnar cells

## Abstract

Columnar cell lesions (CCLs) are recognized precursor lesions of the low nuclear grade breast neoplasia family. CCLs are cystic enlarged terminal duct lobular units with monotonous (monoclonal) columnar-type luminal cells. CCLs without atypia are regarded as benign and CCLs with atypia as true precursor lesions with clonal molecular changes, a certain progression risk, and an association with more advanced lesions. However, reproducibility of designating atypia in CCL is not optimal, and no objective markers of atypia have been identified, although 16q loss seems to be associated with atypical CCLs. Dimorphic (“pale”) cell populations have been described in low nuclear grade ductal carcinoma in situ (DCIS) but not in CCLs and atypical ductal hyperplasia (ADH). Therefore, we searched for pale cells in CCL (*N* = 60), ADH (*N* = 41), and DCIS grade 1 (*N* = 84). Diagnostic criteria were derived from the WHO, and atypia was designated according to the Schnitt criteria. Pale cells occurred in 0% (0/30), 73% (22/30), 56% (23/41), and 76% (64/84) of CCLs without atypia, CCLs with atypia, ADH, and DCIS grade 1, respectively. Pale cells expressed ERα, E-cadherin and p120 and variably cyclin D1, and lacked expression of CK5 and p63. In conclusion, dimorphic “pale” cells occur throughout the low nuclear grade progression spectrum, increasing in frequency with progression. Interestingly, CCL lesions without atypia do not seem to bear showed pale cells, indicating that the presence of pale cells may serve as a diagnostic morphological feature of atypia in CCLs.

## Introduction

Columnar cell lesions (CCLs) of the breast are cystically dilated enlarged terminal duct lobular units lined by columnar luminal cells with uniform, ovoid nuclei and often with apical cytoplasmic blebs or snouts presenting at the luminal surface. The lining is usually one or two cell layers (columnar cell change) although multiple cell layers may be present, usually denoted columnar cell hyperplasia. Intraluminal secretions and microcalcifications are commonly seen [1]. In columnar cell change with atypia, also denoted flat epithelial atypia (FEA), the columnar cells show nuclear atypia of relatively round to ovoid nuclei with or without prominent nucleoli and an increased nuclear/cytoplasmic ratio and/or disturbed nuclear orientation along the basement membrane. A complex architectural pattern (micropapillae, rigid cellular bridges, bars and arcades, or cribriform architecture) necessitates upgrading a CCL to atypical ductal hyperplasia (ADH) or low-grade ductal carcinoma in situ (DCIS) [2, 3].

The diagnosis of atypia in a CCL is of clinical importance. They are both recognized as low-grade preneoplasms of the breast with clonal molecular alterations [4, 5]. Nevertheless, the diagnosis CCL without atypia does not have consequences for treatment because of a low upgrade risk. In contrast, CCLs with atypia are considered true precursor lesions of the low nuclear grade breast cancer family [4–7], with upgrade rates of 5–9% [8, 9] and an association with more advanced lesions (ADH [10–16], DCIS grade 1 [1, 11, 14, 17, 18], lobular neoplasia [10, 12, 14, 16, 19–22], and tubular cancer [7, 17, 19, 20, 23, 24]) in about 20% of patients [25]. This necessitates a discussion about further follow-up and/or treatment in individual patients with atypical CCLs.

However, reproducibility of designating atypia in CCL is generally low. Although O’Malley achieved excellent agreement (multi-rater kappa value 0.83) in diagnosing atypical CCLs after a tutorial in a selected case set [26], other groups found substantially lower kappa values (0.27 and 0.41) [27–29]. Two recent meta-analyses described pooled upgrade rate of pure FEA diagnosed by CNB of 5% and 9% [8, 9]. The difference between these studies is that Wahab et al. also included imaging follow-up, whereas Ferre et al. analyzed only the results of surgical excision. In contrast to the results of these studies based on standardized second opinion, publications without this standard show upgrade rates at the surgical specimen between 0 and 30% for CNB-based diagnosed pure FEAs [30]. This also indicates that the reproducibility of diagnosis of atypia in CCL is not optimal in routine practice. So far, no phenotypical markers of atypia have unfortunately been identified.

Dimorphic cells have been described in the literature in three original publications so far [31–33] Altogether, they have been described in 70 cases (Lefkowitz (1994), 20 cases; Ueno (2018), 50 cases; Koerner (2010), not specified), predominantly in papillary carcinomas [31, 33] and besides in 40 invasive NST carcinomas but also in 10 DCIS cases [32]. The frequency of dimorphic cells in DCIS is however not well-established. Koerner described that careful scrutiny reveals frequent cellular dimorphism in DCIS [33]. Others describe a dimorphic type DCIS as an unusual variant [34, 35].

The cells are characterized by clear cytoplasm-simulating myoepithelial cells but with nuclei similar to those in the adjacent malignant cells, rounded cell borders, and clear cytoplasm in the H&E stain. Several of these articles indicate that dimorphic cell populations are especially seen in low nuclear grade DCIS, which makes it plausible that these “pale cells” would also occur in earlier precursor lesions of the low nuclear grade family. Indeed, our impression was that we regularly encounter pale cells in our practice in low-grade precursor lesions, but dimorphic differentiation has to the best of our knowledge not been described in CCLs and ADH before. This prompted us to systematically retrospectively evaluate the presence of pale cells in a group of ADH and CCL lesions to cover the earliest spectrum of the low nuclear grade precursor lesions, in search of further morphological features of CCLs, especially with regard to the designation “atypia.”

## Material and methods

Slides from 185 formalin-fixed, paraffin-embedded breast tissue samples (biopsies or resections) with CCLs (*N* = 60), ADH (*N* = 41), and DCIS grade 1 (*N* = 84) were collected from the Department of Pathology of the University Medical Center Utrecht between July 2017 and July 2018. CCLs were graded according to the classification described by Schnitt and Vincent-Salomon [3] as CCLs without atypia (*N* = 30) and CCLs with atypia (*N* = 30). Designation of DCIS grade 1 and atypical ductal hyperplasia was assessed by two experienced observers, according to the World Health Organization classification [36, 37]. The presence of co-existing lobular neoplasia (LN) was noted, confirmed by E-cadherin immunohistochemistry when deemed necessary.

A dimorphic cell population was defined as epithelial cells with clear cytoplasm with nuclei similar to those in the adjacent clonal cells, rounded cell borders, and clear cytoplasm in the H&E stain, often located between the luminal and myoepithelial layers, simulating pagetoid spread of LN. The CCLs, ADH, and DCIS lesions were screened for the presence of these pale cells. This was not done in pure LN since pale cells resemble the cells of LN. Routinely performed immunohistochemical stains were screened to identify the expression patterns of pale cells. Since pale cells are often scattered as single cells throughout lesions, E-cadherin stains were especially scrutinized for adjacent pale cells and pale cell groups to pinpoint membrane expression or lack thereof.

## Results

**Fig. 1:**
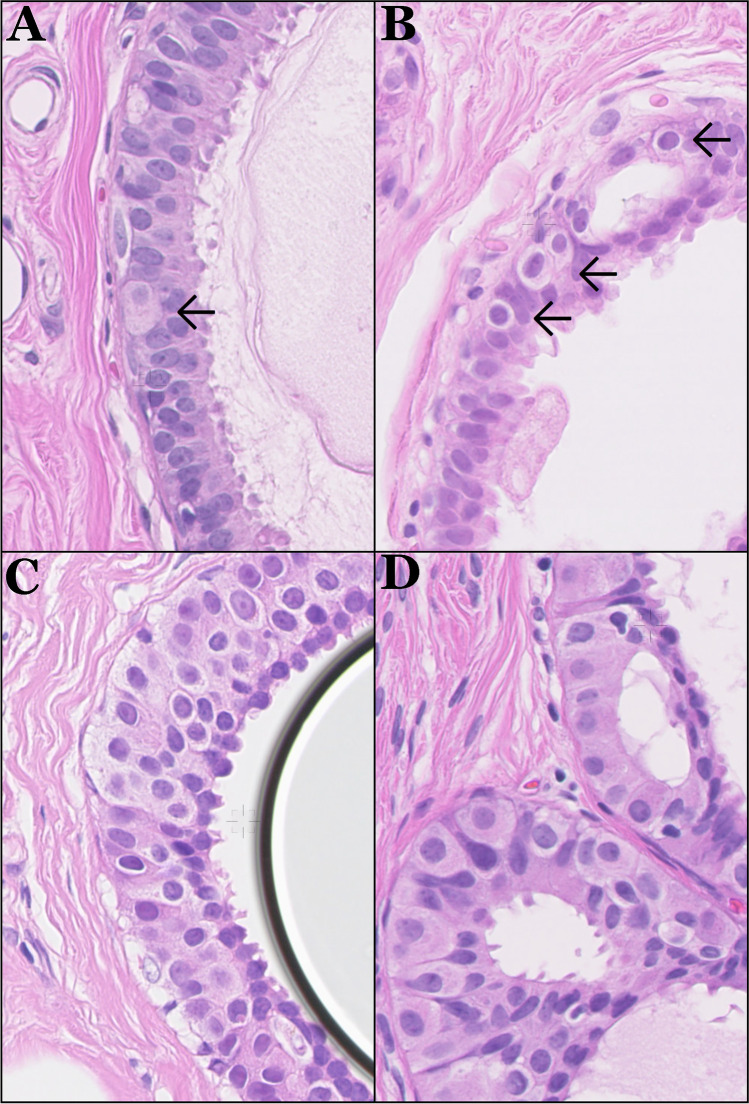
Representative examples of **A**. Single pale cells in CCL with atypia (**B**). Single pale cell in ADH, as well as examples of clonal expansion of pale cells, linear in CCL with atypia (**C**), and forming tubular structures (**D**) in DCIS grade 1

Table 1 shows the frequency of pale cells in the various low nuclear grade breast precursor lesions studied. Pale cells occurred in 0% (0/30), 73% (22/30), 56% (23/41), and 76% (64/84) of CCLs without atypia, CCLs with atypia, ADH, and DCIS grade 1, respectively. Figure 1 shows examples of pale cells in CCLS with atypia, ADH, and DCIS grade 1. In some ADH and DCIS lesions, clusters of pale cells were observed (“clonal expansion”) that rarely formed tubular structures (Fig. 1).

**Table 1:** Frequency of dimorphic (“pale”) cells in different lesions throughout the spectrum of the low nuclear grade breast neoplasia family (*CCL*, columnar cell lesion; *ADH*, atypical ductal hyperplasia; *DCIS*, ductal carcinoma in situ)

Diagnosis	#	# patients	Mean age (range)	# biopsy	# resection	# with pale cells (%)
CCL without atypia	30	29	49.7 (37–70)	25	5	0 (0%)
CCL with atypia	30	27	51.3 (39–71)	21	9	22 (73%)
ADH	41	41	44.5 (40–76)	32	9	23 (56%)
DCIS grade 1	84	67	61.6 (35–84)	35	49	64 (76%)

**Fig. 2:**
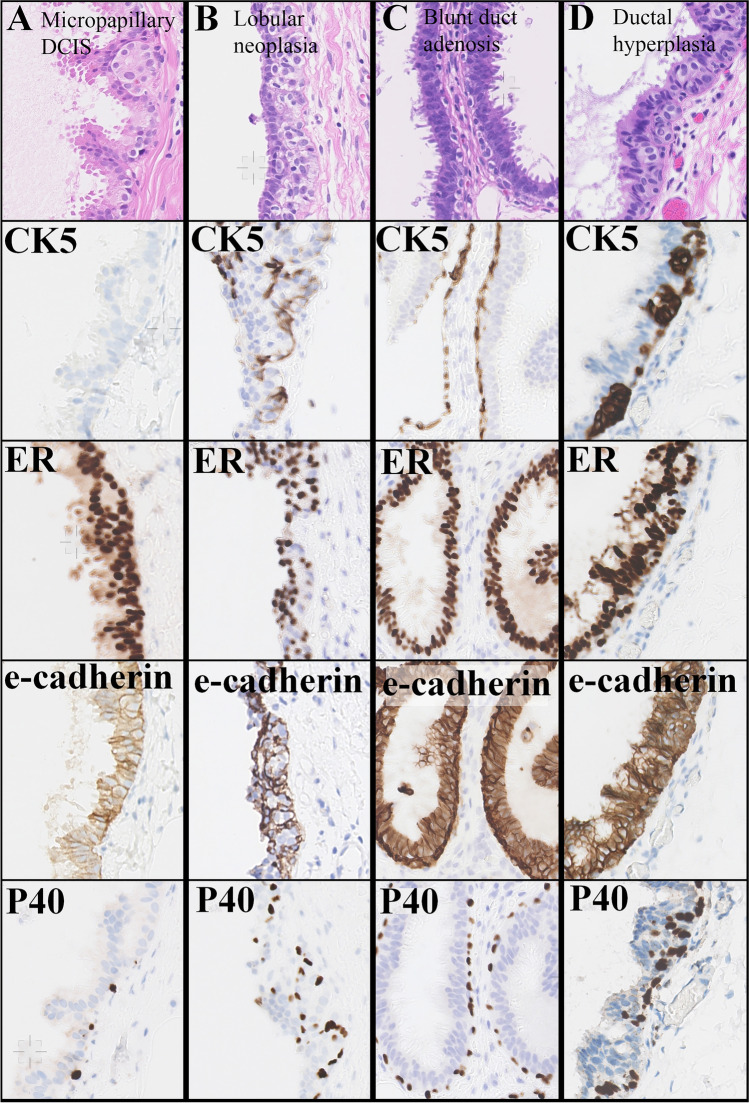
Immunophenotype of pale cells versus its mimics. Column **A**, pale cells in micropapillary ductal carcinoma in situ (column **A**) clonally expressing ERα while lacking CK5 and p40 expression, with normal membrane expression of E-cadherin. Column **B**, pagetoid spread below the pre-existent luminal epithelium of lobular neoplasia cells clonally expressing ERα while lacking CK5 and p40 expression as well as lacking membrane expression of E-cadherin. Column **C**, blunt duct adenosis with prominent myoepithelium that expresses CK5 and p40 while lacking ERα expression. Column **D**, clusters of ductal hyperplastic cells below the pre-existent luminal epithelium expressing CK5 and ERα while lacking p40

**Fig. 3:**
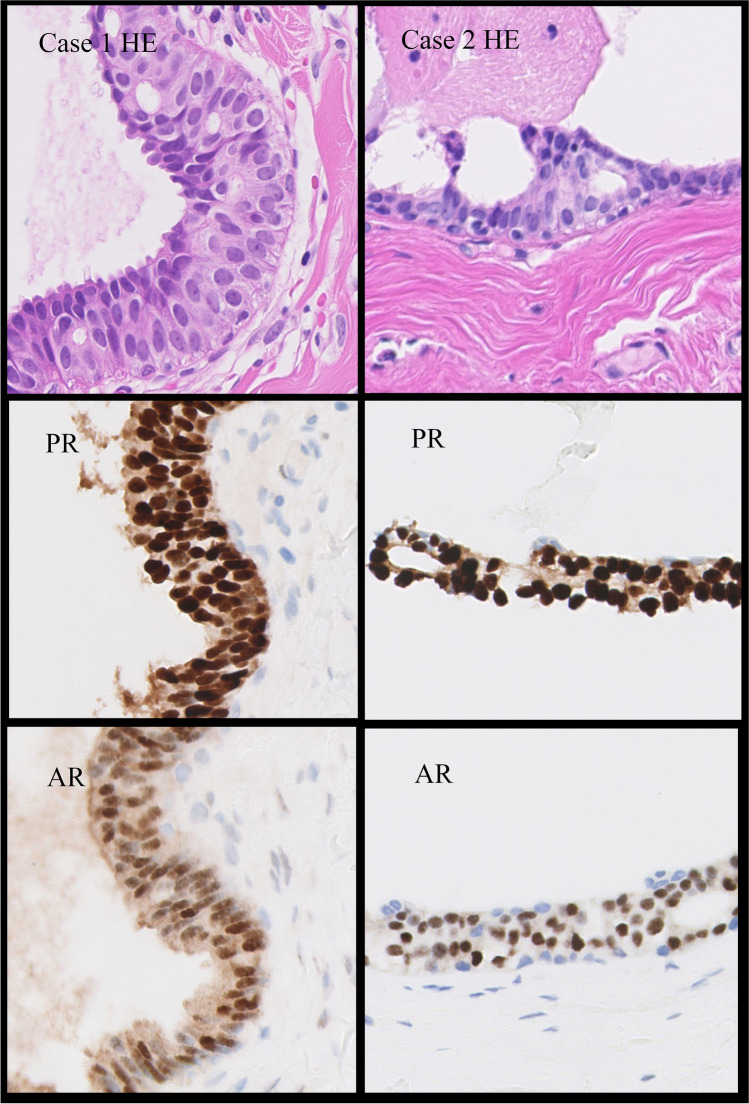
Immunophenotype of pale cells. Case 1, extensive pale cells in atypical ductal hyperplasia clonally expressing PR and AR. Case 2, pagetoid pale cells in micropapillary ductal carcinoma in situ, forming small cribriform structures, clonally expressing PR and AR

Pale cells turned out to be expressing ERα, PR, E-cadherin, AR and p120 and variably cyclin D1, and lacked expression of CK5 and p40 (Fig. 2 and Fig. 3). Figure 2 also shows a comparison of the immunophenotype of pale cells and its mimics. Pagetoid spread of LN below the pre-existent luminal epithelium clonally expressed ERα while lacking CK5, p40 expression, and E-cadherin. Prominent myoepithelium in blunt duct adenosis expressed CK5 and p40 while lacking ERα expression, and clusters of ductal hyperplastic cells below the pre-existent luminal epithelium expressed CK5 and ERα while lacking p40.

## Discussion

Dimorphic “pale” cell populations were first described in papillary DCIS as epithelial cells with clear cytoplasm-simulating myoepithelial cells, but with nuclei similar to those in the adjacent malignant cells, rounded cell borders, and clear cytoplasm in the H&E stain [29], later designated as a feature of low nuclear grade DCIS. We here show that pale cells frequently occur in true low-grade nuclear breast precursor lesions, in 109 of 155 precursor lesions (CCL with atypia 22/30, ADH 23/41, DCIS grade 1 64/84) while being absent in 30 CCL lesions without atypia. This indicates that the presence of pale cells may serve as a diagnostic feature of atypia in CCLs. Pale cells expressed ERα, E-cadherin and p120 and variably cyclin D1, and lacked expression of CK5 and p40.

The biological background of these pale cells is not clear. Theoretically, they could be luminal epithelial cells with a slightly different morphology, scattered apocrine cells, scattered LN cells, neuroendocrine cells, or myoepithelial cells. Since pale cells express ERα and PR, an apocrine origin is unlikely, and the expression of E-cadherin largely rules out LN. The expression of ERα and the lack of CK5 and p40 expression rule out myoepithelial origin [38]. We therefore hypothesize that these pale cells are neoplastic luminal epithelial cells, compatible with the observed expression of ERα and the lack of CK5 expression. We have however no explanation why they morphologically stand out. This requires further molecular studies, e.g., applying single-cell sequencing on microdissected pale cells, but this is yet technically challenging on paraffin-embedded tissue. Perhaps, they are a subclone, as we sometimes see clonal expansion of pale cell-forming groups that start to take over precursor lesions (Fig. 1). This may also explain the previously described dimorphic lesions [31–35]. Further, when lesions are fully comprised of pale cells, they may be hard to designate as “pale,” indicating that the frequency of pale cell lesions reported here may be underestimated. Pale cells have also been described to express AR and BRST2 [32], compatible with their luminal breast origin.

In conclusion, we here describe that dimorphic “pale” cells frequently occur throughout the low nuclear grade breast progression spectrum (CCL with atypia, ADH, DCIS grade 1). Interestingly, CCLs without atypia did not show pale cells, indicating that the presence of pale cells may serve as a diagnostic morphological feature of atypia in CCLs.
